# Effects of Gender and Geographical Origin on the Chemical Composition and Antiradical Activity of *Baccharis myriocephala* and *Baccharis trimera*

**DOI:** 10.3390/foods9101433

**Published:** 2020-10-09

**Authors:** Suzana Struiving, Ana Carolina Mendes Hacke, Edésio Luiz Simionatto, Dilamara Riva Scharf, Cláudia Vargas Klimaczewski, Michele Aparecida Besten, Gustavo Heiden, Aline Augusti Boligon, João Batista Teixeira Rocha, José Carlos Rebuglio Vellosa, Domingos Sávio Nunes, Daniel Granato, Romaiana Picada Pereira

**Affiliations:** 1 Departamento de Química, Universidade Estadual de Ponta Grossa, Av. General Carlos Cavalcanti, 4748-CEP: 84030-900 Ponta Grossa, PR, Brazil; suzanastruiving@gmail.com (S.S.); ana_hacke@hotmail.com (A.C.M.H.); dsnunes@uepg.br (D.S.N.); 2 Laboratório de Cromatografia, Universidade Regional de Blumenau, Rua São Paulo, 3250 CEP: 89030-000 Blumenau, SC, Brazil; edesio@furb.br (E.L.S.); driva@furb.br (D.R.S.); 3 Departamento de Bioquímica e Biologia Molecular, Universidade Federal de Santa Maria, Av. Roraima 1000-CEP: 97105-900 Santa Maria, RS, Brazil; claudiavklima@gmail.com (C.V.K.); joao.rocha@ufsm.br (J.B.T.R.); 4 Instituto Federal do Paraná, Av. Victor Ferreira do Amaral, 306-CEP 82530-230 Curitiba, PR, Brazil; michele.besten@ifpr.edu.br; 5 Embrapa Clima Temperado, Rodovia BR 392 km 78, 403-CEP: 96001-970 Pelotas, RS, Brazil; gustavo.heiden@embrapa.br; 6 Departamento de Farmácia Industrial, Universidade Federal de Santa Maria, Av. Roraima, 1000-CEP: 97105-900 Santa Maria, RS, Brazil; alineboligon@hotmail.com; 7 Departamento de Análises Clínicas e Toxicológicas, Universidade Estadual de Ponta Grossa, Av. General Carlos Cavalcanti, 4748-CEP: 84030-900 Ponta Grossa, PR, Brazil; josevellosa@uepg.br; 8 Natural Resources Institute Finland, Innovative Food System, Production System Unit, Tietotie 2, FI- 02150 Espoo, Finland

**Keywords:** *carqueja*, principal component analysis, gas chromatography–mass spectrometry with flame-ionization detection (GC-MS-FID), high performance liquid chromatography (HPLC), essential oil, polyphenols

## Abstract

This study aimed to characterize and compare essential oils and ethyl acetate fractions obtained in basic and acidic conditions from both male and female *Baccharis* species (*Baccharis myriocephala* and *Baccharis trimera*) from two different Brazilian regions. Samples were characterized according to their chemical compositions and antiradical activity by 2,2-diphenyl-1-picrylhydrazyl (DPPH) and 2,2′-azino-bis(3-ethylbenzothiazoline-6-sulfonic acid) (ABTS) assays. Principal component analysis (PCA) provided a clear separation regarding the chemical composition of essential oils from the samples obtained from different regions by using gas chromatography–mass spectrometry with flame-ionization detection (GC-MS-FID). PCA also revealed that gender and region of plant collections did not influence the chemical composition and antiradical activity of ethyl acetate fractions, which was corroborated with hierarchical cluster analysis (HCA) data. High performance liquid chromatography with diode-array detector (HPLC-DAD) identified significant quantities of flavonoids and phenolic acids in the fractions obtained in basic and acidic fractions, respectively. The obtained results clearly demonstrated that the geographical region of plant collection influenced the chemical composition of essential oils from the studied *Baccharis* species. Moreover, the obtained fractions were constituted by several antiradical compounds, which reinforced the usage of these species in folk medicine.

## 1. Introduction

*Baccharis myriocephala* DC. and *Baccharis trimera* (Less.) DC., Asteraceae, belong to *Baccharis* sect. Caulopterae and are commonly known as “*carqueja*” [1]. They are native plants found in South America and used in folk medicine to treat hepatic and gastrointestinal disorders. Further, they are also used as a natural alternative for body weight loss [2,3]. Additionally, these plants are popularly used to treat anemia, hypertension, fever, and rheumatism [4,5]. The main constituents of *Baccharis* genus are phenolic and terpenoid compounds that can contribute to its biological properties, such as anticancer, antidiabetic, anti-inflammatory, antifungal, and antimicrobial [6,7]. The mentioned species are frequently confused in the popular field, being used for the same therapeutic purposes. Thus, it is important to obtain phytochemical data to clarify the differentiation in the chemical composition of these species, contributing to their safer usage.

Several studies have been conducted in order to verify the chemical composition and pharmacological effects of *Baccharis* genus extracts and fractions [6,8,9]. Phytochemical analyses have indicated the presence of a wide range of bioactive compounds, mainly polyphenols such as isoquercetin, apigenin, quercetin, rutin, and hispidulin [10]. These compounds are well-known as a consequence of their free-radical scavenging properties and for inhibiting lipid peroxidation [11].

Oxidative stress has been considered one of the main reasons for the progression of several chronic diseases in humans. The high levels of reactive oxygen species (ROS) might provoke oxidation of proteins and lipids, leading to biological disorders [12]. In recent years, several studies have proved that extracts from *Baccharis* genus exhibit remarkable antioxidant activity [7,13,14]. For instance, Paiva et al. [13] showed that the hydroalcoholic extract from *B. trimera* leaves can improve the oxidative stress resistance by increasing survival rate and by reducing ROS levels under oxidative stress conditions in vivo. In another work, Pádua et al. [14] observed that this same extract could reduce the release of ROS in neutrophils by using both in vitro and in vivo models. In addition, De Oliveira et al. [7] found that the ethyl acetate extract from *B. trimera* showed the highest antioxidant activity comparing to aqueous and dichloromethane extracts and also suggested that the anti-inflammatory activity exhibited by the plant was mainly due to the presence of phenolic compounds.

The chemical composition of *Baccharis* genus essential oils is characterized by the predominance of carquejol, carquejil acetate, and sesquiterpenes [15]. However, some researchers found that spathulenol and limonene are the most abundant compounds [16,17]. The differences between these studies can be attributed to the geographical location and environmental factors, which have an influence on the chemical composition of *Baccharis* essential oils [18,19]. Interestingly, some studies have also shown differences in the chemical composition of oils from male and female specimens of *Baccharis*. Some authors have assumed that these species are dioecious and require more attention since gender can influence its biological properties [6,18,20,21]. For example, the female specimen allocates more energy and nutrients to reproduction than male plants. In this way, the female plant has reduced growth, low nutritional status, and are less attacked by herbivorous insects than male plants [21,22]. This behavior is due to the fact that female species tend to grow slowly when compared to male, which favors the increasing defense against pathogens [21,22].

Considering the usage of *Baccharis* specimens in folk medicine and the fact that the chemical composition of the plant is influenced by species-specific, environmental, and geographical conditions, the aim of this work was to evaluate the possible differences in the chemical composition and antiradical activity of both male and female specimens of *B. myriocephala* and *B. trimera* from distinct Brazilian regions. To the best of our knowledge, this is the first report regarding the investigation of the influence of gender and geographical origin in the antiradical activity of ethyl acetate fractions (acidic and basic) from the *Baccharis* species.

## 2. Materials and Methods

### 2.1. Chemicals

All solvents and chemicals used in this study were of analytical grade. Folin–Ciocalteu’s phenol reagent (FCR), 2,2-diphenyl-1-picrylhydrazyl (DPPH), 2,2′-azino-bis(3-ethylbenzothiazoline-6-sulfonic acid) (ABTS), quercetin (QER), quercitrin, rutin, kaempferol, ascorbic acid (AA), luteolin, catechin, epicatechin, and potassium hexacyanoferrate(III) K_3_[Fe(CN)_6_] were purchased from Sigma Chemical Co (St. Louis, MO, USA). Acetonitrile, methanol, phosphoric acid, gallic acid (GA), chlorogenic acid, caffeic acid, and ellagic acid were acquired from Merck (Darmstadt, Germany). Trichloroacetic acid (TCA), sodium sulphate (Na_2_SO_4_), potassium persulfate (K_2_S_2_O_8_), ferric chloride (FeCl_3_), sodium carbonate (Na_2_CO_3_), potassium acetate (CH_3_COOK), and aluminum chloride (AlCl_3_) were obtained from Vetec (Rio de Janeiro, Brazil). Deionized water was used to prepare all aqueous solutions.

### 2.2. Plant Materials

Male and female specimens of *carquejas* were collected in the cities of Ponta Grossa (25°08′ 30” S and 49° 58′ 51” W), Paraná state, Brazil (male: PRM and female: PRF), Paulínia (22° 45′ 40” S and 47° 09′ 15” W), and São Paulo state, Brazil (male: SPM and female: SPF). The samples were identified as *B. trimera* and *B. myriocephala*, according to Heiden et al. [23]. Voucher specimens were deposited in the Herbarium from Embrapa Clima Temperado (ECT), Pelotas, Rio Grande do Sul state, Brazil, under the numbers ECT0005576 and ECT0005575, respectively. Prior to the extraction procedures, the plant samples were dried at room temperature for 72 h, impurities were carefully removed, and the plants were stored in a freezer at −18 °C until further fractionation procedures. Aerial parts of *carquejas* were also commercially acquired in specialized stores in metropolitan areas of Paraná (CoPR) and São Paulo (CoSP) to make comparisons with those collected.

### 2.3. Essential Oil Obtention and Extracts Preparations

Approximately 100 g of each sample (PRM, PRF, SPM, SPF, CoPR, and CoSP) were submitted to a hydrodistillation process which was performed for 2 h by using a Clevenger-type apparatus. The essential oils were collected in ethyl ether and the organic layers were dried employing anhydrous Na_2_SO_4_. Then, the obtained solutions were filtered and the solvents were removed under reduced pressure to obtain the essential oils named PRMV and PRFV from Paraná state and male and female specimens, respectively. Samples from São Paulo state were named SPMV and SPFV for male and female specimens, respectively, and commercial samples from PR and SP states were named PRCoV and SPCoV, respectively

The remaining aqueous residues after the hydrodistillation processes were reserved for extractions procedures [24]. These aqueous residues were partitioned with chloroform (2 × 250 mL) to obtain the fractions named PRCoCl, SPCoCl, PRMCl, PRFCl, SPMCl, and SPFCl. Then, the pH value of the aqueous phases was adjusted (pH 8) using 2 mol L^−1^ NaOH, followed by extraction with ethyl acetate (2 × 250 mL) to obtain the fractions PRCo8, SPCo8, PRM8, PRF8, SPM8, and SPF8. Afterwards, the remaining aqueous phases were acidified with concentrated HCl (pH 4) and extracted with ethyl acetate (2 × 250 mL) to obtain the fractions named PRCo4, SPCo4, PRM4, PRF4, SPM4, and SPF4. All organic phases were dried with anhydrous Na_2_SO_4_, filtered, and the solvents were removed under vacuum using a rotary evaporator.

### 2.4. Essential Oil Analysis

The analysis of *B. trimera* and *B. myriocephala* essential oils was carried out by employing a Varian^®^ CP-3800 gas chromatograph, equipped with Saturn^®^ GC-MS Workstation Software v. 5.51. The instrument was operated in El mode at 70 eV with a mass scan range of 40–650 m/z at a sample rate of 1.0 scan s^−1^. A capillary column CP-Sil long-8 CB Low Bleed/MS 30 m with a diameter of 0.25 mm and a 0.25 mm film was used. The injector and the interface temperatures were kept at 250 °C and 240 °C, respectively. Helium was used as the carrier gas at a flow rate of 5 mL min^−1^. The temperature was set to increase as follows: 50 °C during the first minute, and then 3 °C min^−1^ up to 240 °C (split ratio: 1/50). The relative retention indexes were determined using different alkanes (C8-C18) analyzed under the same conditions as those used for the essential oil samples. In order to validate the system and ensure the reliability of the calculated retention rates, standards were used for α-pinene, β-pinene, linalool, limonene, spathulenol, caryophyllene, and viridiflorol.

The relative composition of essential oil was determined on a Shimadzu 14B gas chromatograph with a flame ionization detection system detector (GC-FID) and an OV-5 column (30 m × 0.25 mm × 0.25 mm). Nitrogen was employed as the carrier gas at a constant pressure of 80 kPa (split ratio: 1/150). An aliquot of 1 µL of the oil diluted in ethyl ether was used for injection. The detector and injector temperatures were kept at 300 °C and 250 °C, respectively. The initial column temperature was 50 °C with a heating rate of 5 °C min^−1^ until 270 °C, where the temperature was maintained for 8 min. The analysis of each essential oil sample was carried out in triplicate.

### 2.5. Determination of Total Phenolic Content

The total phenolic content was measured as described by Singleton et al. [25]. Five microliters of each fraction were mixed with 10% (*v/v*) FCR (100 µL) and 7.5% (*w/v*) Na_2_CO_3_ aqueous solution (125 µL). After an incubation for 15 min at 45 °C the absorbance value was measured at 765 nm in a microplate reader. An analytical curve of gallic acid standard was constructed for the determination of total phenolic content for each fraction. The results were expressed as mg of gallic acid equivalent (GAE) g^−1^ f fraction.

### 2.6. Determination of Total Flavonoid Content

Total flavonoid content was measured as described by Kosalec et al. [26] by complexation with Al(III) ions. Five microliters of each fraction were mixed with 10% (*w/v*) AlCl_3_ (4 µL) and CH_3_COOK 0.1 mmol L^−1^ (4 µL) aqueous solutions and methanol to give a final volume of 225 µL. The absorbance value was measured at 420 nm by using a microplate reader. An analytical curve of quercetin standard was constructed for the determination of total flavonoid content for each fraction. The results were expressed as mg of quercetin equivalent (QE) g^−1^ of fraction.

### 2.7. High Performance Liquid Chromatography (HPLC) Analysis

The ethyl acetate fractions of *B. myriocephala* and *B. trimera* were analyzed by high performance liquid chromatography, as described by Boligon et al. [27] with slight modifications. Analyses were carried out by using a Shimadzu^®^ Prominence Auto Sampler (SIL-20A) HPLC system (Shimadzu, Kyoto, Japan), equipped with Shimadzu^®^ LC-20AT reciprocating pumps connected to a DGU 20A5 degasser with a CBM 20A integrator and SPD-M20A diode array detector. A LC solution 1.22 SP1 software was used for data processing. Reverse phase chromatographic analyses were performed under gradient conditions using a Phenomenex C18 column (4.6 mm × 150 mm) filled with 5 μm diameter particles. The mobile phase consisted of a combination of solvent A (methanol: water; 9:1; *v/v*) adjusted to pH 2.0 with phosphoric acid and solvent B (acetonitrile: water: methanol; 60:20:20; *v/v/v*). Next, 40 mL of each sample were separately injected into the chromatograph at a flow rate of 0.6 mL min^−1^ and the analyses were performed following a linear solvent gradient: 0 min, 100% A; 10 min 30% A; 20 min, 40% A; 50 min, 0% A; held at 0% A for 15 min. The system was equilibrated for five minutes with 100% A before and after each injection. Prior to chromatography analysis *B. myriocephala* and *B. trimera* ethyl acetate fractions (10 mg mL^−1^) and mobile phase were filtered through 0.45 μm membrane filter (Millipore^®^), and degassed in an ultrasonic bath. The stock solutions of polyphenols standards (gallic acid, catechin, epicatechin, ellagic acid, chlorogenic acid, caffeic acid, quercitrin, quercetin, luteolin, kaempferol, and rutin) were prepared in the mobile phase with concentrations ranging from 0.020 mg mL^−1^ to 0.500 mg mL^−1^. The quantification of these components was performed by integrating the peaks using the external standard method at 257 nm for gallic acid, 280 nm for catechin/epicatechin, 325 nm for ellagic, chlorogenic/caffeic acids, and 365 for quercetin, quercitrin, rutin, kaempferol, and luteolin. The retention times and DAD spectra (200–600 nm) of standards were compared to the results obtained for the studied fractions in order to confirm the observed chromatographic peaks.

### 2.8. Antioxidant Activity Determination

#### 2.8.1. Reducing Power Assay

The reducing power capacity of ethyl acetate fractions from *B. myriocephala* and *B. trimera* was evaluated according to the method reported by Mathew and Abraham [28]. The reaction was performed in 0.2 mol L^−1^ phosphate buffer (pH 6.6), 1% (*w/v*) K_3_[Fe(CN)_6_], and the fractions (1–100 µg mL^−1^) to give a final volume of 148 µL. The obtained mixtures were incubated at 50 °C for 20 min and 10% (*w/v*) TCA (86 µL) was added. The tubes were centrifuged at 3000 rpm for 10 min and 86 µL of the upper layer solution was mixed with distilled water (86 µL). Finally, 28 µL of 0.1% (*w/v*) FeCl_3_ was added and the absorbance value was measured at 700 nm in spectrophotometer. Ascorbic acid was used as a positive control.

#### 2.8.2. DPPH and ABTS Scavenging Assays

The DPPH scavenging capability of each fraction was assessed by using the method described by Brand-Williams et al. [29], employing ascorbic acid as a positive control. The fractions (1–100 µg mL^−1^) were mixed with 120 µmol L^−1^ DPPH ethanolic solution and the mixtures were incubated for 30 min at room temperature in the dark. The absorbance values were measured at 518 nm in a microplate reader.

The ABTS scavenging capacity of each fraction was assessed according to the method described by Pellegrini et al. [30], which employed ascorbic acid as a positive control. The ABTS radical cation was obtained by reacting ABTS solution with 140 mmol L^−1^ K_2_S_2_O_7_, followed by incubation in the dark for 12–16 h before use. For the analysis, the fractions (1–100 µg mL^−1^) were mixed with 10 mmol L^−1^ phosphate buffer (pH 7.4) and 7 mmol L^−1^ ABTS^+^ solutions. The obtained mixtures were incubated for 30 min at room temperature in the dark. The absorbance values were measured at 734 nm in a microplate reader.

The antiradical activity for each sample was measured as a decrease in the absorbance of DPPH/ABTS radicals and were calculated using Equation (1):

% inhibition = 100 − [(Abs_sample_ − Abs_blank_) × 100/Abs_control_] (1)

where Abs_sample_ is the absorbance of radicals in the presence of the sample; Abs_blank_ is the absorbance of the samples without adding radicals; and Abs_control_ is the absorbance of pure radical solutions. IC_50_ (inhibitory concentration) values were calculated from % inhibition for each fraction.

### 2.9. Statistical Analysis

The results of chemical composition and antiradical activity were expressed as mean ± standard deviation (SD) obtained from three individual experiments carried out in triplicate. One-way analysisof variance (ANOVA) was performed followed by Tukey’s test to check for differences (*p* < 0.05) between samples. Aiming to characterize the male and the female *B. myriocephala* and *B. trimera* specimens, principal component analysis (PCA) was used in autoscaled data [31]. Statistical analysis was performed using GraphPad Prism v. 5.0 software and Statistica 7 (StatSoft, Tulsa, OK, USA).

## 3. Results and Discussion

The results of GC-MS-FID analyses of the essential oils obtained from the leaves of female and male specimens of *carquejas* and from the two commercial samples from São Paulo and Paraná states are displayed in Table 1. According to the literature, sesquiterpenoids and monoterpenoids are the predominant compounds in *Baccharis* genus essential oils [15,19,21]. In general, it was observed that the samples with the highest concentrations of oxygenated monoterpenes and oxygenated sesquiterpenes were collected in Paraná (PRMV and PRFV) and São Paulo (SPFV and SPM) states, respectively. Interestingly, the commercial sample PRCoV exhibited higher amounts of oxygenated monoterpenes compared to SPCoV.

**Figure 1:**
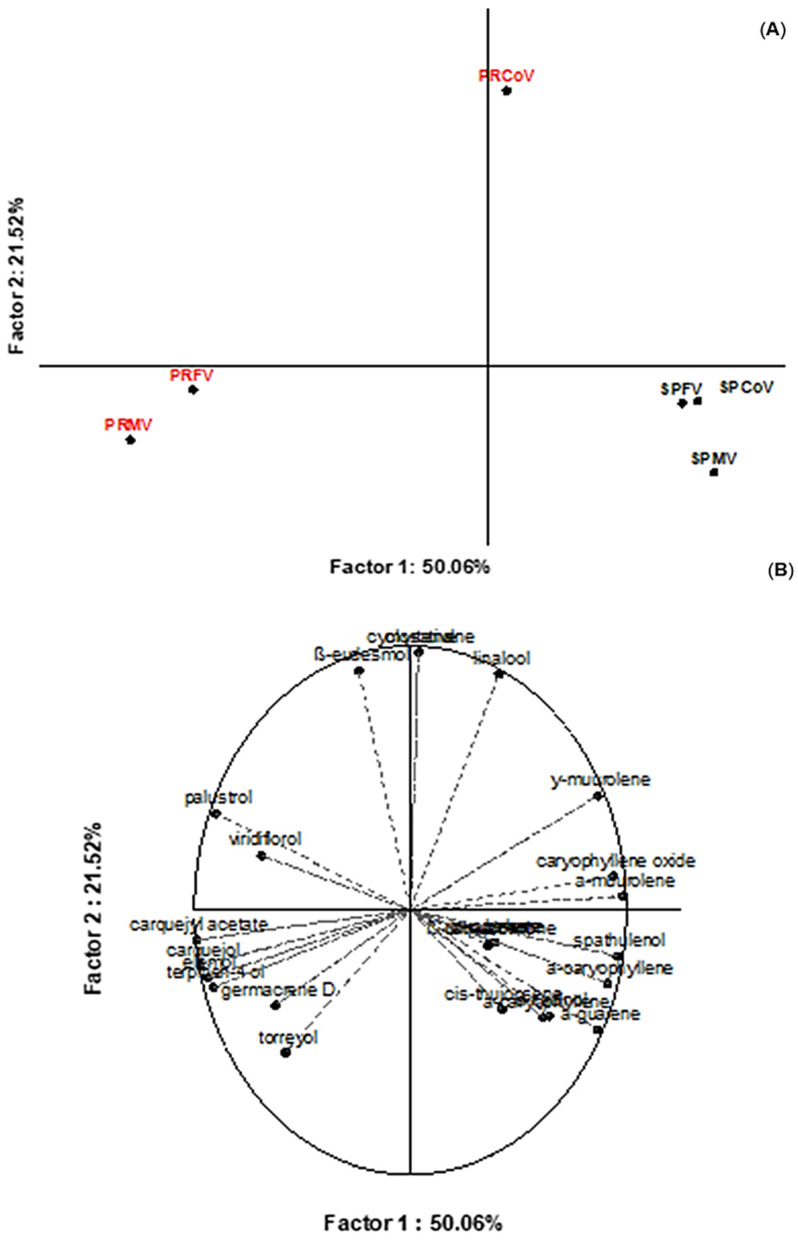
Projection of *B. myriocephala* and *B. trimera* samples according to the levels of volatile compounds in the essential oils (**A**) and the factor loadings (**B**). The red color represents the essential oils from Paraná State and the black color is related to the essential oil from São Paulo State. V = essential oil, Co = commercial samples, F = female specimens, M = male specimens, SP = São Paulo state, and PR = Paraná state.

Figure 1 demonstrates the variation in the chemical composition of *B. myriocephala* and *B. trimera* essential oils from male and female specimens and from two commercial samples from Paraná and São Paulo. The similarity of essential oils was evaluated via PCA, which revealed a strong inter-relationship and explained up to 71% of total variance in the chemical composition of essential oils (Figure 1). The concentrations of the compounds in the essential oils obtained from leaves collected in the same regions were similar and gender did not have a significant impact on the selected chemical compounds. PRMV and PRFV samples presented carquejyl acetate, palustrol, and carquejol as the major components. On the other hand, the SPFV, SPMV, and SPCoV samples presented spathulenol, followed by caryophyllene oxide and β-caryophyllene as their major constituents. Besides that, it is possible to observe that the volatiles extracted from the commercial sample PRCoV were mainly spathulenol followed by carquejyl acetate and palustrol (Table 1).

Our results are in agreement with those reported by Besten et al. [20], which showed clear similarities between the metabolism of male and female specimens of five different *Baccharis* species. In another study, Besten et al. [32] verified that there was no significant difference in the chemical composition of *B. trimera* essential oils from Southern Brazil. However, our results suggest that the chemical composition of essential oils from *Baccharis* genus was clearly influenced by the species identified and the geographical region where the plant was collected. Simões-Pires et al. [15] suggested that carquejyl acetate is a chemomarker for the identification of *B. trimera*, since relative concentrations up to 69% (*w/w*) regarding this compound were observed in the essential oil of the mentioned species. Nevertheless, Silva et al. [19] did not identify this compound in their samples and it was suggested that the variation pattern in the essential oils could be due to the existence of chemotypes for *B. trimera*, that have not been identified and described yet for this species. These behaviors agree with our results, since greater proportions of carquejyl acetate were obtained from *B. trimera* samples collected in Paraná state, whereas minor levels of this compound were found in *B. myriocephala* from São Paulo state. Furthermore, it is known that the chemical composition of essential oils varies according to climatic and geographic conditions [19]. Our results are in-line with those reported by Trombin-Souza et al. [17], which verified that *B. myriocephala* is constituted by minor proportions of carquejyl acetate.

Although the aqueous residue from the hydrodistillation process is usually considered as a waste by essential oil industries, some authors [33,34,35] have demonstrated that this phase is rich in compounds that present biological activities, such as antiradical capability. In order to extract such bioactive compounds, the aqueous residues were partitioned with different organic solvents.

It is well known that medicinal plants are constituted by different classes of chemical compounds which have different polarities, so the solvent choice displays an important role to separate their constituents. Firstly, chloroform extractions were performed in order to remove the least polar compounds from the aqueous phases. After these extractions, the pH value of the residual medium was changed to 8, followed by extractions with ethyl acetate to favor the extraction of flavonoids. After that, the pH value of the residual aqueous phases was changed to 4, and then new extractions with ethyl acetate were performed in order to extract phenolic acids [33]. As expected, the total contents of phenolic compounds in the chloroform fractions were the lowest compared to those obtained from ethyl acetate (Table 2). This can be justified by the fact that these compounds are more soluble in solvents with a higher polarity. On the other hand, the ethyl acetate fractions exhibited variations on the total content of phenolic and flavonoid compounds. It is well known from the literature that several factors may contribute to changes in the concentrations of secondary metabolites in plants, such as seasonality, temperature, altitude, and collection conditions [36]. This can explain the differences in the phenolic and flavonoid levels observed in our study when comparing the different samples analyzed.

Phenolic compounds were determined in ethyl acetate fractions by using HPLC. The obtained results show that 11 different phenolic compounds (7 flavonoids and 4 phenolic acids) were detected in relatively high concentrations in the studied fractions (Table 3; Supplementary Materials Figure S1), namely gallic acid (retention time-tR 10.07 min, peak 1), catechin (tR = 15.83 min, peak 2), chlorogenic acid (tR = 19.98 min, peak 3), caffeic acid (tR = 23.85 min, peak 4), ellagic acid (tR = 27.31 min, peak 5), epicatechin (tR = 29.64 min, peak 6), rutin (tR = 36.19 min, peak 7), quercitrin (tR = 40.79 min, peak 8), quercetin (tR = 43.80 min, peak 9), kaempferol (tR = 44.31 min, peak 10), and luteolin (tR = 52.10 min, peak 11). The chromatograms (Supplementary Materials Figure S1) and Table 3 also revealed that gallic, chlorogenic, caffeic, and ellagic acids were the major compounds in the fractions obtained at pH 4, whereas the flavonoids, kaempferol, catechin, epicatechin, quercitrin, quercetin, rutin, and luteolin appeared to be the major constituents in those obtained at pH 8.

DPPH and ABTS scavenging assays are widely employed for the evaluation of the antiradical activity of plant extracts. In our study, it was demonstrated that *B. myriocephala* and *B. trimera* ethyl acetate fractions have antiradical effects by DPPH, which means that the compounds present in the samples exhibited hydrogen donating ability (Table 2). The HPLC results (Table 3) showed that these fractions exhibited significant quantities of non-flavonoids which are good DPPH scavengers, such as gallic and caffeic acids [37]. The ABTS assay was also employed for the evaluation of the antiradical activity and, in agreement with DPPH results, fractions were composed by compounds which were good ABTS scavengers. This indicated the electron-donating properties of the compounds present in the fractions. Hence, the obtained results evidence the antiradical activity of the *B. myriocephala* and *B. trimera* fractions, as well as the hydrogen and electron-donating abilities of phenolic compounds, which are related to their chemical structures.

Previous studies have verified a good correlation between the polyphenol content and the antiradical activity by DPPH and ABTS methods [38,39]. However, the differences observed in the antiradical activity comparing these two methods can be explained by the fact that some antiradical compounds present in the fractions may have a slower reaction rate or are inert to these radicals.

**Figure 2:**
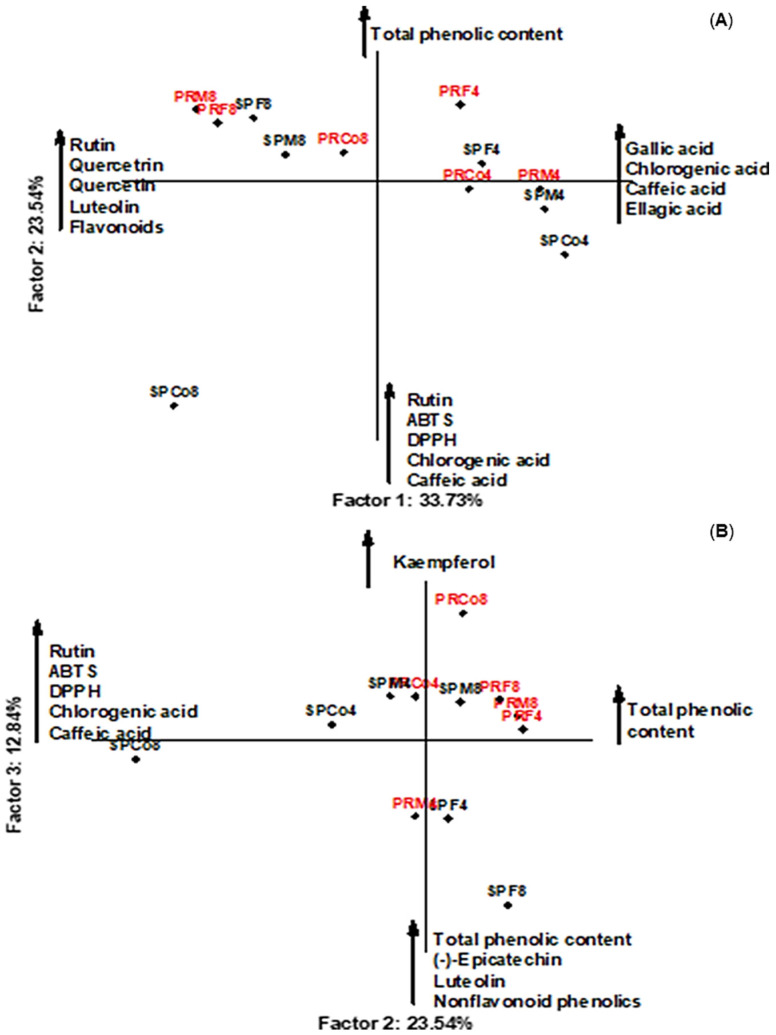
Principal component analysis (PCA) of *B. trimera* and *B. myriocephala* ethyl acetate fractions according to the levels of bioactive compounds and antioxidant activity: PC1 vs. PC2 (**A**) and PC2 vs. PC3 (**B**). Co = commercial samples, F = female specimens, M = male specimens, SP = São Paulo state, and PR = Paraná state. The presented numbers are related to the pH conditions used to obtain each fraction.

PCA was employed in order to evaluate the phenolic compounds quantified by HPLC, total contents of phenolics, flavonoids, and non-flavonoids, as well as the antiradical activity measured by DPPH and ABTS assays (Figure 2). Samples were separated along the first principal component (PC1) by differences on the concentrations of rutin, quercitrin, quercetin, luteolin, total content of flavonoids, and phenolic acids (gallic, chlorogenic, caffeic, and ellagic acids). PC1 explained up to 33.73% and PC2 explained up to 23.54% of total variance. Thus, the presented two-dimensional graph was able to explain 57.27% of the variability in the experimental data (Figure 2A). Besides that, PC3 explained 12.84% and was able to explain 36.38% of the variability in the experimental data (Figure 2B). Consequently, the first three principal components—i.e., PC1, PC2, and PC3—explained 70.11% of total variance. It is noteworthy that all ethyl acetate fractions from *B. trimera* and *B. myriocephala* obtained at pH 4 were located in the 1st and 2nd quadrants (right side), and the fractions at pH 8 were located in the 3rd and 4th quadrants (left side) (Figure 2A). Furthermore, the total phenolic content was not affected by the region of collection, analyzed specimens nor the gender, and the chemical compositions were similar between the collected and commercial samples from Paraná and São Paulo states.

**Figure 3:**
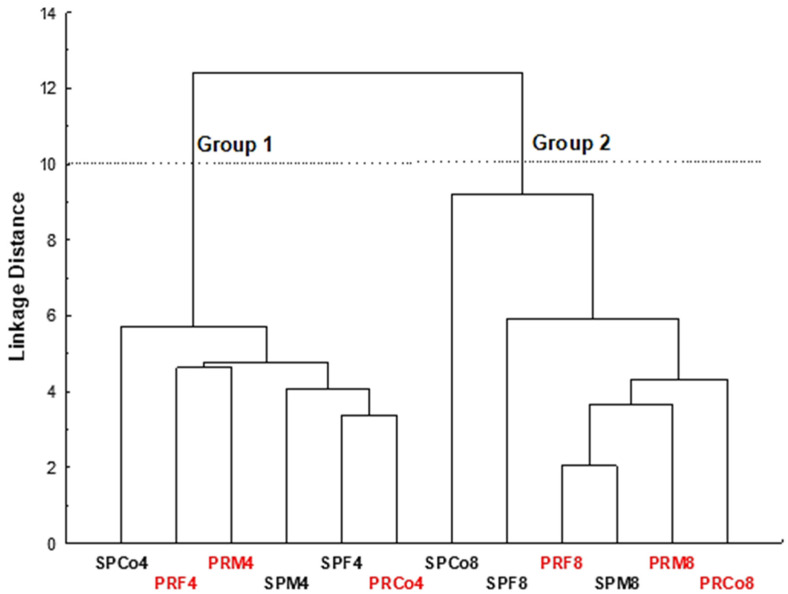
Hierarchical cluster analysis (HCA) of *B. trimera* and *B. myriocephala* ethyl acetate fractions according to the levels of polyphenol and antiradical activity. Co = commercial samples, F = female specimens, M = male specimens SP = São Paulo state, and PR = Paraná state. The presented numbers are related to the pH conditions used to obtain each fraction.

The similarities between the ethyl acetate fractions of collected and commercial samples were analyzed using a hierarchical cluster analysis (HCA), in which two clusters were identified (Figure 3). These clusters corroborated the results from the PCA (Figure 2A). Cluster 1 contains the ethyl acetate fractions with the highest content of phenolic acids (those obtained at pH 4) and the lowest content of flavonoids. On the other hand, cluster 2 shows the ethyl acetate fractions with the highest content of flavonoids (those obtained at pH 8). It can be observed that the fractions obtained from male and female *Baccharis* specimens were grouped in both clusters 1 and 2. Thus, the results suggested again that there was no significant difference in the chemical composition of ethyl acetate fractions of male and female specimens. Differently, it was verified that the essential oils showed differences in their chemical composition for *B. myriocephala* and *B. trimera* species.

**Figure 4:**
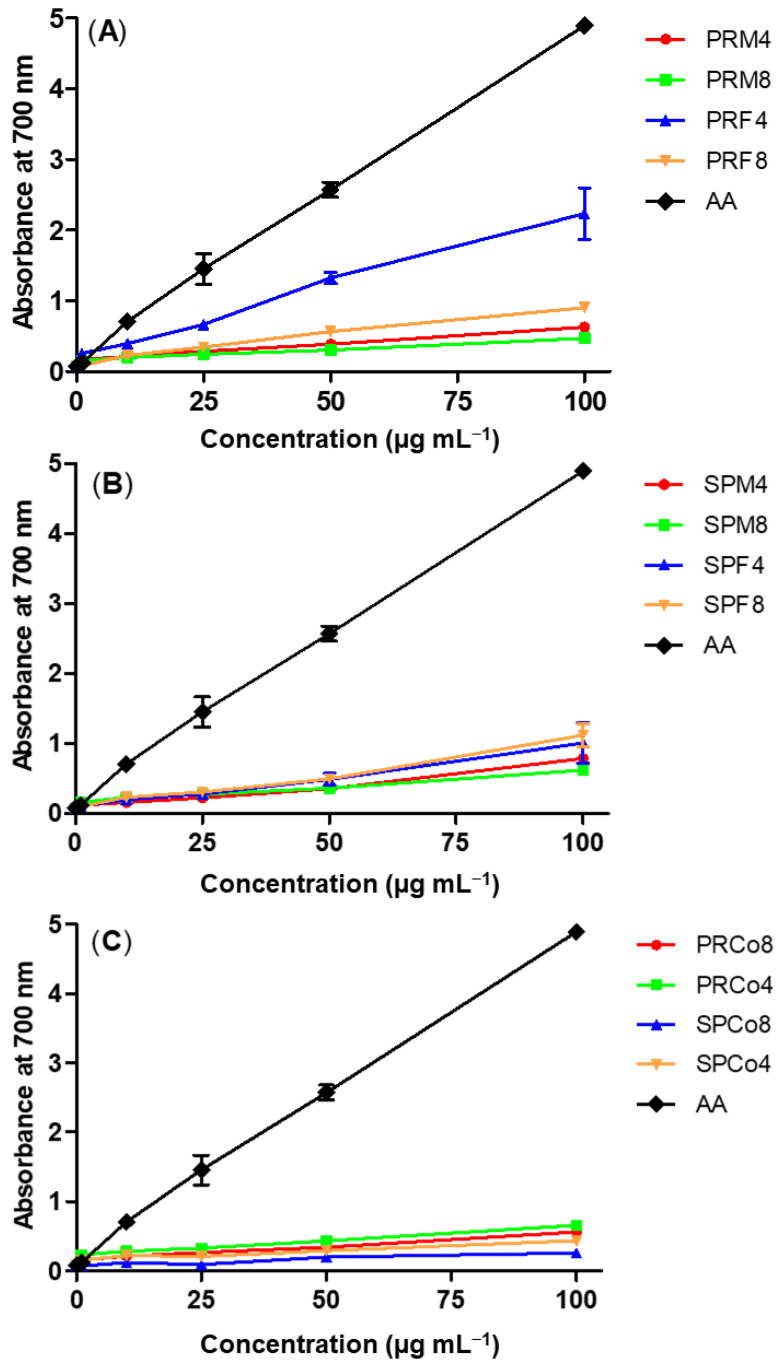
Reducing power assay of ethyl acetate fractions of (**A**) *B. trimera* and (**B**) *B. myriocephala* and (**C**) commercial samples from Paraná and São Paulo states. The data show mean ± standard deviation (SD) values as an average of 3 independent experiments performed in duplicate. Co = commercial samples, F = female specimens, M = male specimens SP = São Paulo state and PR = Paraná state. The presented numbers are related to the pH conditions used to obtain each fraction.

Figure 4 demonstrates the reducing power results of assays performed with ethyl acetate fractions from male and female specimens of *B. trimera* and *B. myriocephala* from Paraná and São Paulo states. The reducing power is directly associated with the antioxidant activity of a compound, due to the ability to reduce [Fe(CN)_6_]^3−^ to [Fe(CN)_6_]^4−^ to form Prussian Blue complex salt after reaction with Fe(III) ions from FeCl_3_ [28]. As illustrated in Figure 4, all fractions exhibited antioxidant activity and this property was similar between the samples due to the analogous increases in the absorbance values. Just like ABTS^+^ method, the reducing power assay activity indicates the electron-donating properties of the bioactive compounds present in the ethyl acetate fractions. By these two distinct methods, it was possible to prove the great electron-donating capability of the components present in the ethyl acetate fractions from two *Baccharis* species. The reducing ability of a compound is due to the presence of phenolic hydroxyl groups in its chemical structure, as well as due to the resonance between the free electron pair on the phenolic oxygen and the benzene ring. Compounds with reducing power ability indicate that they are able to react with free radicals stabilizing and blocking radical chain reactions [40].

Using a two-way ANOVA and considering gender and location as factors, the isolated effects and the significance of the interaction gender*location were calculated (Table 4) regarding the phenolic composition and antiradical activity of *B. myriocephala* and *B. trimera* samples. According to the results, only the DPPH data and (-)-epicatechin content was significant between locations, whereas there was a significant difference (*p* < 0.05) between genders and for the interaction gender*location for the DPPH data. These results may be associated with the use of different samples for the same factor, which diminishes the variation between samples from male/female specimens and São Paulo/Paraná states.

## 4. Conclusions

In the present study, we demonstrated that the chemical composition of essential oils of *B. trimera* and *B. myriocephala* from two different regions of Brazil were similar between genders, but significantly differed regarding species identity. On the other hand, these two parameters did not appear to influence the total content of polyphenols and antiradical activity of ethyl acetate fractions obtained in acidic and basic conditions. Our work demonstrated that these fractions were rich in polyphenols, which justified their antioxidant activity. However, in vivo studies should be done to verify the safety of these plants’ fractions.

## Figures and Tables

**Table 1:** Relative composition of the identified components in the analyses of the essential from *B. myriocephala* and *B. trimera* leaves.

Components	RI ^a^	RI ^b^	PRCoV (%)	SPCoV (%)	PRFV (%)	SPFV (%)	PRMV (%)	SPMV (%)
Linalool	1096	1097	2.62 ± 0.37	0.77 ± 0.16	n.d	0.86 ± 0.12	n.d	0.62 ± 0.11
Carquejol	1151	1151	0.86 ± 0.05	n.d	6.17 ± 0.38	n.d	8.89 ± 0.18	n.d
Terpinen-4-ol	1177	1174	n.d	n.d	0.96 ± 0.07	n.d	1.93 ± 0.24	n.d
α-terpineol	1189	1185	n.d	n.d	n.d	0.55 ± 0.05	n.d	n.d
Myrtenol	1194	1200	1.00 ± 0.10	n.d	n.d	n.d	n.d	n.d
Carquejyl acetate	1292	1292	11.33 ± 4.31	n.d	53.04 ± 2.33	0.47 ± 0.41	53.41 ± 1.84	n.d
α-cubebene	1351	1349	n.d	0.86 ± 0.38	n.d	n.d	n.d	n.d
Cyclosativene	1368	1368	0.98 ± 0.12	n.d	n.d	n.d	n.d	n.d
α-copaene	1376	1373	n.d	0.65 ± 0.24	n.d	n.d	n.d	n.d
β-damascenone	1386	1383	n.d	n.d	n.d	0.57 ± 0.09	n.d	n.d
β-caryophyllene	1418	1409	0.87 ± 0.33	4.34 ± 0.17	n.d	3.11 ± 0.25	n.d	3.05 ± 0.79
α-guaiene	1439	1444	n.d	0.86 ± 0.24	n.d	0.81 ± 0.12	n.d	0.73 ± 0.10
α-caryophyllene	1454	1448	n.d	n.d	n.d	0.88 ± 0.18	n.d	0.90 ± 0.11
γ-muurolene	1477	1475	0.92 ± 0.35	0.77 ± 0.67	n.d	0.93 ± 0.54	n.d	0.59 ± 0.31
Germacrene D	1480	1485	n.d	n.d	0.61 ± 0.06	1.19 ± 0.16	2.14 ± 0.19	n.d
α-muurolene	1499	1499	1.21 ± 0.45	1.99 ± 0.06	n.d	1.70 ± 0.29	n.d	1.59 ± 0.22
Elemol	1547	1538	n.d	n.d	0.89 ± 0.14	n.d	0.73 ± 0.11	n.d
Palustrol	1557	1556	8.22 ± 2.72	3.73 ± 0.24	9.27 ± 1.06	0.98 ± 0.26	10.32 ± 0.29	n.d
Spathulenol	1576	1568	17.67 ± 3.51	27.45 ± 4.14	2.89 ± 0.15	33.75 ± 6.40	2.21 ± 0.08	40.79 ± 1.52
Caryophyllene oxide	1581	1571	6.93 ± 1.33	5.84 ± 3.09	n.d	9.04 ± 5.32	n.d	9.36 ± 0.57
Viridiflorol	1590	1590	2.49 ± 0.14	1.99 ± 0.39	2.70 ± 0.24	2.77 ± 1.21	3.23 ± 0.47	0.90 ± 0.06
Cis-thujopsene	1628	1626	n.d	n.d	n.d	n.d	n.d	3.64 ± 0.95
Torreyol	1645	1641	n.d	n.d	1.25 ± 0.13	n.d	1.29 ± 0.07	1.30 ± 0.36
β-eudesmol	1649	1650	0.92 ± 0.45	n.d	0.47 ± 0.14	n.d	n.d	n.d
α-cadinol	1653	1652	nd	0.89 ± 0.23	n.d	n.d	n.d	0.92 ± 0.31

n.d = not detected by gas chromatography–mass spectrometry (GC-MS) or the content was below 0.10%. Values are expressed as mean ± standard deviations (SD) of experiments performed in triplicate.RI ^a^: relative retention indices were calculated by using the non-polar columns CP-Sil-8 (GC-MS) and an OV-5 for gas chromatograph with flame-ionization detection (GC-FID) and the n-alkane series C10-C30; RI ^b^: relative retention indices calculated by using the non-polar column DB-5. V = essential oil, Co = commercial samples, F = female specimens, M = male specimens, SP = São Paulo state, and PR = Paraná state.

**Table 2:** Total phenolic, flavonoid, and non-flavonoid content, as well as the antiradical activity of *B. myriocephala* and *B. trimera* fractions.

Fractions	Total Phenolics (mg GAE g^−1^) *	Total Flavonoids (mg QE g^−1^) *	Total Non-flavonoids (mg g^−1^) *	ABTS IC_50_ (µg mL^−1^) *	DPPH IC_50_ (µg mL^−1^) *
AA	-	-	-	7.24 ± 1.00 a	13.20 ± 0.24 a
SPCoCl	45.21 ± 3.65 c	1.45 ± 0.02 f	43.76 ± 4.76 c	16.50 ± 0.93 b	57.01 ± 0.67 b
PRCoCl	42.78 ± 1.27 c	2.46 ± 0.09 e	40.32 ± 1.70 c	47.90 ± 2.39 d	57.36 ± 0.60 b
SPCo4	76.51 ± 3.97 b	2.69 ± 0.02 e	73.82 ± 5.77 a	14.24 ± 3.29 b	53.29 ± 6.01 b
PRCo4	64.22 ± 4.64 b	2.21 ± 0.10 e	62.01 ± 6.08 b	6.79 ± 0.88 a	10.45 ± 4.60 a
SPCo8	33.35 ± 0.79 d	2.22 ± 0.13 e	31.13 ± 1.13 d	75.94 ± 6.89 d	60.50 ± 2.79 b
PrCo8	48.50 ± 1.02 c	3.42 ± 0.06 d	45.08 ± 1.24 c	6.07 ± 1.00 a	12.34 ± 4.34 a
SPFCl	35.16 ± 0.67 d	1.61 ± 0.02 f	33.55 ± 0.89 d	> 100	74.80 ± 6.21 c
PRFCl	35.68 ± 5.49 d	2.13 ± 0.07 e	33.55 ± 7.75 d	26.36 ± 2.20 c	58.36 ± 0.52 b
SPF4	73.08 ± 7.02 b	2.03 ± 0.01 e	71.05 ± 10.03 a	10.50 ± 1.82 b	12.58 ± 4.80 a
PRF4	59.79 ± 1.30 c	4.13 ± 0.46 c	55.66 ± 2.42 b	6.34 ± 1.13 a	11.86 ± 3.25 a
SPF8	85.99 ± 6.03 a	2.98 ± 0.07 d	83.01 ± 8.37 a	12.01 ± 1.32 b	18.94 ± 2.14 a
PRF8	51.12 ± 0.51 c	6.02 ± 0.08 b	45.10 ± 0.83 c	11.64 ± 2.28 b	10.64 ± 6.22 a
PRMCl	34.87 ± 0.95 d	2.23 ± 0.04 e	32.64 ± 1.29 d	13.64 ± 2.11 b	56.62 ± 0.31 b
SPMCl	31.06 ± 1.08 d	1.77 ± 0.11 e	29.29 ± 1.28 d	89.20 ± 8.90 e	63.37 ± 5.34 b
SPM4	45.97 ± 0.35 c	1.96 ± 0.04 e	44.01 ± 0.46 c	11.69 ± 1.95 b	14.75 ± 0.63 a
PRM4	46.88 ± 4.00 c	2.76 ± 0.10 e	44.12 ± 5.30 c	10.95 ± 2.28 b	11.86 ± 3.25 a
PRM8	68.50 ± 0.60 b	10.90 ± 0.11 a	57.60 ± 0.92 b	13.07 ± 0.70 b	11.24 ± 3.60 a
SPM8	54.59 ± 1.14 c	3.07 ± 0.05 d	51.52 ± 1.58 b	9.01 ± 0.23 b	16.24 ± 1.06 a

* Data are expressed as mean ± standard deviations (SD) (*n* = 3) of experiments performed in triplicate. Averages followed by different letters differ significantly at *p* < 0.05. GAE = gallic acid equivalent; QE = quercetin equivalent; AA = ascorbic acid; IC_50_ = inhibitory concentration. Co = commercial samples, F = female specimens, M = male specimens, SP = São Paulo state, and PR = Paraná state. The presented numbers are related to the pH conditions used to obtain each fraction.

**Table 3:** Concentrations of polyphenols of *B. myriocephala* and *B. trimera* fractions.

Compounds	PRCo8	PRCo4	SPCo8	SPCo4	SPF8	SPF4	PRM8	PRM4	SPM8	SPM4	PRF8	PRF4
	(mg g−^1^) *	(mg g−^1^) *	(mg g−^1^) *	(mg g−^1^) *	(mg g−^1^) *	(mg g−^1^) *	(mg g−^1^) *	(mg g−^1^) *	(mg g−^1^) *	(mg g−^1^) *	(mg g−^1^) *	(mg g−^1^) *
Gallic acid	1.97 ± 0.02 f	1.95 ± 0.01 d	1.83 ± 0.01 e	3.72 ± 0.03 c	0.76 ± 0.01 e	2.11 ± 0.03 b	0.73 ± 0.02 e	1.59 ± 0.01 d	0.73 ± 0.04 f	1.86 ± 0.03 d	0.78 ± 0.01 e	1.81 ± 0.01 c
Catechin	0.18 ± 0.01 h	1.03 ± 0.03 e	0.79 ± 0.01 g	0.76 ± 0.01 g	0.74 ± 0.03 e	0.79 ± 0.02 e	0.70 ± 0.01 e	1.54 ± 0.01 d	0.69 ± 0.01 f	0.65 ± 0.02 f	0.75 ± 0.02 e	1.49 ± 0.01 d
Chlorogenic acid	2.45 ± 0.01 e	3.78 ± 0.01 a	3.56 ± 0.02 c	3.89 ± 0.01 b	1.93 ± 0.01 c	3.07 ± 0.02 a	1.98 ± 0.03 c	4.12 ± 0.03 a	2.14 ± 0.01 c	2.51 ± 0.01 b	1.64 ± 0.01 d	1.86 ± 0.03 c
Caffeic acid	1.96 ± 0.03 f	3.81 ± 0.01 a	3.61 ± 0.04 c	3.91 ± 0.02 b	1.97 ± 0.01 c	3.04 ± 0.01 a	2.03 ± 0.01 c	2.09 ± 0.02 b	2.17 ± 0.03 c	4.03 ± 0.04 a	1.60 ± 0.01 d	2.37 ± 0.04 b
Ellagic acid	3.02 ± 0.02 d	3.39 ± 0.02 c	1.82 ± 0.01 e	3.27 ± 0.01 d	0.78 ± 0.04 e	3.10 ± 0.04 a	2.06 ± 0.01 c	4.05 ± 0.01 a	2.03 ± 0.02 d	4.09 ± 0.01 a	0.69 ± 0.03 e	1.89 ± 0.01 c
Epicatechin	0.21 ± 0.01 h	0.24 ± 0.03 f	1.45 ± 0.01 f	0.80 ± 0.04 g	1.43± 0.02 d	1.35 ± 0.01 d	0.69 ± 0.04 e	1.61 ± 0.02 d	0.71 ± 0.01 f	1.38 ± 0.01 e	0.71 ± 0.04 e	0.11 ± 0.03 f
Rutin	0.38 ± 0.02 g	1.07 ± 0.01 e	3.51 ± 0.03 c	1.09 ± 0.01 f	2.09 ± 0.01 c	0.81 ± 0.01 e	1.90 ± 0.01 d	0.74 ± 0.04 e	1.54 ± 0.03 e	0.67 ± 0.02 f	1.58 ± 0.01 d	0.62 ± 0.02 e
Quercitrin	3.76 ± 0.01 b	3.35 ± 0.04 c	4.73 ± 0.02 b	2.03 ± 0.03 e	3.87 ± 0.01 a	2.96 ± 0.03 a	5.26 ± 0.03 a	1.83 ± 0.01 c	4.05 ± 0.02 b	1.98 ± 0.03 c	3.96 ± 0.01 b	1.51 ± 0.01 d
Quercetin	3.47 ± 0.03 c	3.87 ± 0.01 a	5.11 ± 0.01 a	2.05 ± 0.03 e	3.86 ± 0.02 a	3.01 ± 0.01 a	4.15 ± 0.01 b	2.15 ± 0.01 b	4.23 ± 0.01 a	1.85 ± 0.04 d	4.01 ± 0.02 b	2.40 ± 0.01 b
Kaempferol	4.85 ± 0.03 a	3.62 ± 0.01 b	4.74± 0.04 b	4.34 ± 0.01 a	2.07 ± 0.03 c	1.32 ± 0.01 d	4.19 ± 0.03 b	0.81 ± 0.02 e	4.25 ± 0.01 a	2.59 ± 0.03 b	4.39 ± 0.03 a	2.58 ± 0.04 a
Luteolin	1.96 ± 0.02 f	0.98 ± 0.01 e	2.69 ± 0.01 d	0.75 ± 0.02 g	2.93 ± 0.01 b	1.57 ± 0.04 c	1.97 ± 0.02 c	1.63 ± 0.01 d	1.50 ± 0.02 e	0.67 ± 0.01 f	1.85 ± 0.01 c	1.47 ± 0.03 d

* Data are expressed as mean ± SD (*n* = 3). Averages followed by different letters differ statistically at *p* < 0.05. Co = commercial samples, F = female specimens, M = male specimens, SP = São Paulo state, and PR = Paraná state. The presented numbers are related to the pH conditions used to obtain each fraction.

**Table 4:** Two-way ANOVA to check for differences between gender and location and the interaction between these factors on the phenolic composition and antiradical activity of *B. myriocephala* and *B. trimera* samples.

Factor	Gender	Location	Gender*Location
Gallic acid	0.193	0.482	0.811
(+)-Catechin	0.665	0.402	0.544
Chlorogenic acid	0.158	0.668	0.421
Caffeic acid	0.264	0.150	0.909
Ellagic acid	0.258	0.998	0.753
(-)-Epicatechin	0.413	0.049	0.203
Rutin	0.898	0.342	0.450
Quercetrin	0.941	0.993	0.861
Quercetin	0.863	0.990	0.981
Kaempferol	0.192	0.807	0.357
Luteolin	0.662	0.930	0.520
Total phenolics	0.455	0.592	0.375
Total flavonoids	0.542	0.142	0.560
Non-flavonoid phenolics	0.412	0.412	0.346
ABTS	0.437	0.252	0.288
DPPH	<0.001	<0.001	<0.001

## Supplementary Materials

The following are available online at https://www.mdpi.com/2304-8158/9/10/1433/s1, Figure S1: High performance liquid chromatography (HPLC) profiles of *B. myriocephala* and *B. trimera* fractions: (**A**) PRCo8; (**B**) PRCo4; (**C**) SPCo8; (**D**) SPCo4; (**E**) SPF8; (**F**) SPF4; (**G**) PRM8; (**H**) PRM4; (**I**) SPM8; (**J**) SPM4; (**K**) PRF8; and (**L**) PRF4. Gallic acid (peak 1), catechin (peak 2), chlorogenic acid (peak 3), caffeic acid (peak 4), ellagic acid (peak 5), epicatechin (peak 6), rutin (peak 7), quercitrin (peak 8), quercetin (peak 9), kaempferol (peak 10), and luteolin (peak 11).
