# Supplementary material for: Effects of Gender and Geographical Origin on the Chemical Composition and Antiradical Activity of Baccharis myriocephala and Baccharis trimera
Source: Foods. 2020 Oct 9;9(10):1433. doi: 10.3390/foods9101433 (PMC7601893; doi:10.3390/foods9101433)
Supplement: Supplementary file 1 [file foods-09-01433-s001.pdf]

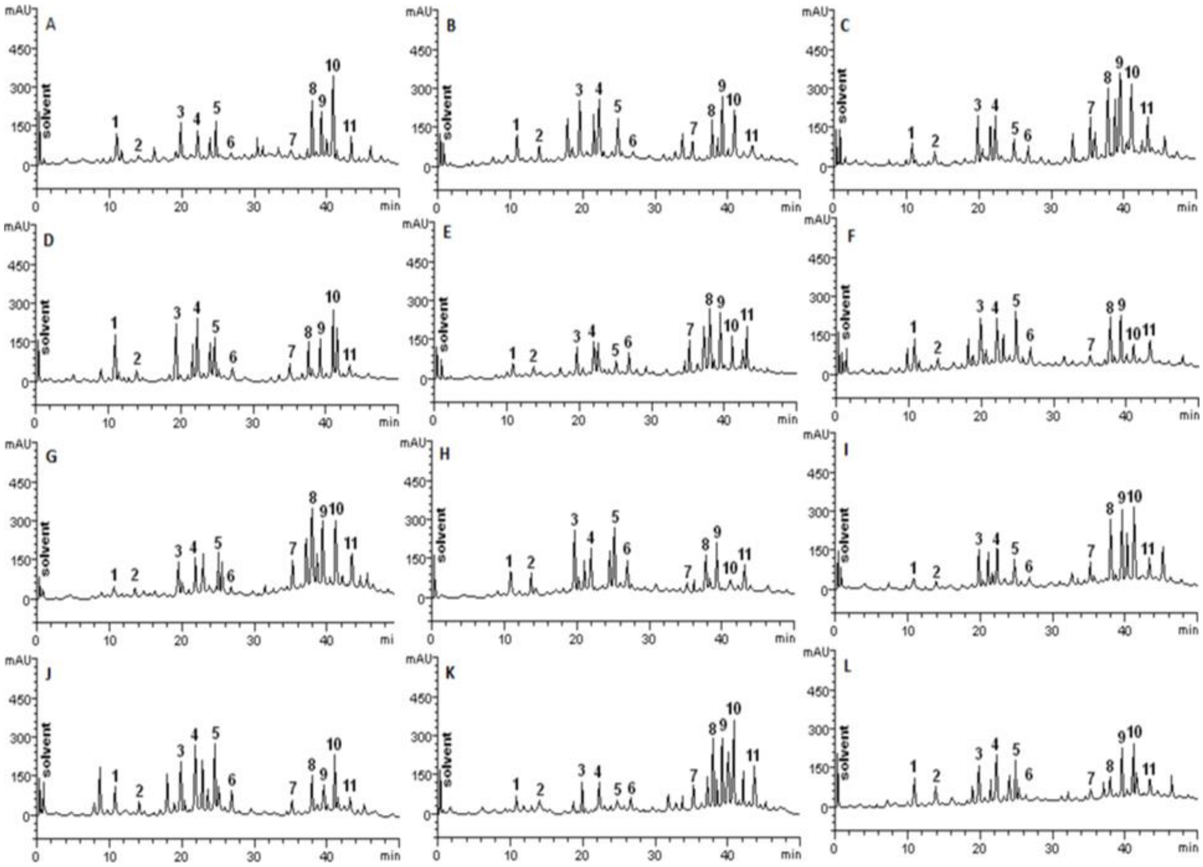

Figure S1. High performance liquid chromatography (HPLC) profiles of *B. myriocephala* and *B. trimera* fractions: (**A**) PRCo8; (**B**) PRCo4; (**C**) SPCo8; (**D**) SPCo4; (**E**) SPF8; (**F**) SPF4; (**G**) PRM8; (**H**) PRM4; (**I**) SPM8; (**J**) SPM4; (**K**) PRF8 and (**L**) PRF4. Gallic acid (peak 1), catechin (peak 2), chlorogenic acid (peak 3), caffeic acid (peak 4), ellagic acid (peak 5), epicatechin (peak 6), rutin (peak 7), quercitrin (peak 8), quercetin (peak 9), kaempferol (peak 10) and luteolin (peak 11).
